# Impact of Isoorientin on Metabolic Activity and Lipid Accumulation in Differentiated Adipocytes

**DOI:** 10.3390/molecules25081773

**Published:** 2020-04-13

**Authors:** Khanyisani Ziqubu, Christo J. F. Muller, Phiwayinkosi V. Dludla, Sinenhlanhla X. H. Mthembu, Nnini Obonye, Johan Louw, Abidemi P. Kappo, Sonia Silvestri, Patrick Orlando, Luca Tiano, Sithandiwe E. Mazibuko-Mbeje

**Affiliations:** 1 Biomedical Research and Innovation Platform, South African Medical Research Council, Tygerberg 7505, South Africa; khanyisani.ziqubu@mrc.ac.za (K.Z.); pdludla@mrc.ac.za (P.V.D.); sinenhlanhla.mthembu@mrc.ac.za (S.X.H.M.); nnini.obonye@mrc.ac.za (N.O.); johan.louw@mrc.ac.za (J.L.); 2 Department of Biochemistry and Microbiology, University of Zululand, KwaDlangezwa 3886, South Africa; akappo@uj.ac.za; 3 Division of Medical Physiology, Faculty of Health Sciences, Stellenbosch University, Tygerberg 7505, South Africa; 4 Department of Life and Environmental Sciences, Polytechnic University of Marche, 60131 Ancona, Italy; s.silvestri@univpm.it (S.S.); p.orlando@univpm.it (P.O.); l.tiano@univpm.it (L.T.); 5 Department of Biochemistry, Faculty of Science, University of Johannesburg, Kingsway Campus, Auckland Park 2006, South Africa; 6 Department of Biochemistry, Faculty of Natural and Agricultural Sciences, North West University, Mafikeng Campus, Mmabatho 2735, South Africa

**Keywords:** isoorientin, obesity, metabolic activity, lipid accumulation, 3T3-L1 adipocytes

## Abstract

The current study explored the effect of isoorientin on the metabolic activity and lipid accumulation in fully differentiated 3T3-L1 adipocytes. To achieve this, the 3T3-L1 pre-adipocytes were differentiated for eight days and treated with various concentrations of isoorientin (0.1–100 μM) for four hours. Subsequently, the metabolic activity, lipid accumulation, and mitochondrial respiration were assessed. Furthermore, to unravel the molecular mechanisms that might elucidate the bioactivity of isoorientin, protein expression of the genes involved in insulin signaling and energy expenditure, such as AKT and AMPK, were investigated. The results showed that isoorientin, at different doses, could block lipid storage and enhance glycerol release, with a concomitant improvement of the metabolic activity and mitochondrial function. Although the observed beneficial effects of isoorientin on these cultured 3T3-L1 adipocytes were not consistent at all concentrations, it was clear that doses between 1 and 10 μM were most effective compared to the untreated control. Moreover, the activity of isoorientin was comparable to tested positive controls of CL-316,2431, isoproterenol, insulin, and metformin. Mechanistically, protein expression of AKT and AMPK, was enhanced with isoorientin exposure, suggesting their partial role in modulating lipid metabolism and mitochondrial biogenesis. Indeed, our results showed that isoorientin has the ability to enhance mitochondrial respiration, as we observed an increase in the ATP and oxygen consumption rate. Therefore, we concluded that isoorientin has a potential to impact mitochondrial activity, lipid metabolism and energy expenditure using an in vitro experimental model of obesity.

## 1. Introduction

Natural products are considered a safe approach in treating metabolic syndrome [1], and their therapeutic potential against obesity has been extensively reviewed [2,3,4]. Of interest is isoorientin, a *C*-glycosyl flavonoid, which is found in rooibos, among other plants, and which displays strong ameliorative effects on obesity-linked complications [5,6]. Isoorientin, also referred to as homoorientin, is a *C*-glucosyl flavone that was first isolated by Koeppen and colleagues in 1962 from *Aspalathus linearis*, a South African indigenous plant, which is widely consumed as herbal tea (rooibos) [7,8]. Of general interest, is evidence showing that plant extracts rich in this glucosyl flavone display enhanced anti-obesity properties, including amelioration of various metabolic complications [9,10,11]. Several studies conducted over the past few years in our laboratory, consistently advocate the ameliorative effect of the rooibos extracts containing isoorientin, against various metabolic disorders in different experimental models [12,13,14]. For example, our previous study [13] showed that an extract of fermented rooibos could block adipogenesis and affect adipocyte metabolism in cultured 3T3-L1 adipocytes. This is especially important, since although the Food and Drug Administration (FDA) approved drugs for obesity, such as orlistat and others, are effective, a major concern exists regarding their long-term use and associated adverse side effects [15]. Such limitations have raised interest in expanding research into the use of natural products as therapeutic agents to prevent obesity or its associated complications. Although some studies conducted over the years showed isoorientin, or plants rich in this natural compound, can ameliorate obesity-related complications, such as oxidative stress, inflammation, hyperlipidemia, and insulin resistance [5,16,17,18,19,20], there is still relatively little information about its impact as a pure compound on obesity.

In this study, we investigated a concentration-dependent effect of isoorientin on metabolic activity and lipid accumulation in fully differentiated 3T3-L1 adipocytes. We further explored some of the mechanisms involved in this process, especially those implicated in insulin signaling and energy metabolism. 

## 2. Results 

### 2.1. Isoorientin Enhanced ATP Production and Glucose Uptake without Inducing Any Cytotoxicity in Matured 3T3-L1 Adipocytes

**Figure 1:**
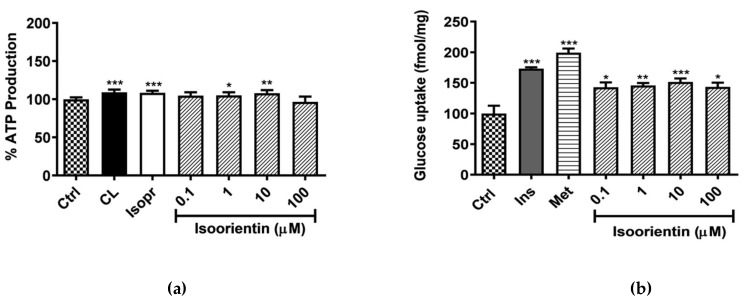
Isoorientin increased ATP production (**a**) and glucose uptake (**b**) in fully differentiated 3T3-L1 adipocytes. Mature 3T3-L1 adipocytes were treated with or without positive control CL-316,243 (CL), isoproterenol (Isopr), insulin (Ins), metformin (Met) used at 1 µM and various doses of isoorientin (0.1, 1, 10, and 100 µM) for 4 hours. Results are expressed as mean ± SEM of three independent experiments. * *p* < 0.05, ** *p* ˂ 0.01, *** *p* ˂ 0.001 versus normal control (Ctrl).

The ATP assay was used to assess metabolic activity of cells after exposure to isoorientin. The results showed that that both controls (CL and Isopr) enhanced ATP content by 9% (*p* < 0.0001) (Figure 1a). Consistently, isoorientin at 1 and 10 µM, enhanced ATP content by 5% and 8% (*p* < 0.05 and *p* < 0.0001) respectively, thereby enhancing metabolic activity of cells (Figure 1a). Interestingly, all the doses of isoorientin tested did not show cytotoxicity, as there was no significant decrease in ATP production when compared to the control.

In terms of glucose metabolism, the two antidiabetic agents, insulin (1 µM) and metformin (1 µM), showed enhanced effects to promote glucose uptake by 73% and 99% (*p* < 0.0001), respectively, when compared to the control. All tested concentrations of isoorientin also significantly increased glucose uptake when compared to the control (Figure 1b). Interestingly, the positive effects of isoorientin were not dose-dependent, with the concentration of 10 µM showing more potency, and a more than 52% (*p* < 0.001) improvement in glucose uptake, when compared to the experimental control (Figure 1b).

### 2.2. Isoorientin Reduced Intracellular Lipid Accumulation and Enhanced Lipolysis in Matured 3T3-L1 Adipocytes

**Figure 2:**
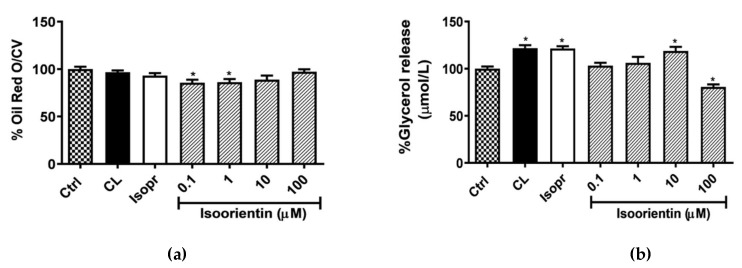
Isoorientin reduced lipid accumulation (**a**) and increased glycerol release (**b**) in 3T3-L1 adipocytes. Matured 3T3-L1 adipocytes were treated with or without positive CL-316,243 (CL), isoproterenol (Isopr) used at 1 µM and various doses of isoorientin (0.1, 1, 10, and 100 µM) for 4 hours; subsequently, lipid accumulation was measured with Oil Red O and confirmed with glycerol release assay. Results are expressed as mean ± SEM of three independent experiments. * *p* < 0.05 versus normal control (Ctrl).

The quantification and analysis of ORO was performed to determine lipid accumulation in matured 3T3-L1 adipocytes. The positive controls, CL and Isopr, had no noticeable effect after four hours of culture, while isoorientin reduced lipid accumulation, with a significant reduction of 14% at both 0.1 and 1 µM (*p* < 0.05) respectively (Figure 2a). The reduction in lipid accumulation was accompanied by increased glycerol release from the dose of 1 μM, which is the end product for lipolysis (Figure 2b). However, only the isoorientin dose of 10 μM significantly reduced cellular lipid content (*p* < 0.05). 

### 2.3. Isoorientin Improved Mitochondrial Respiration in Matured 3T3-L1 Adipocytes

**Figure 3:**
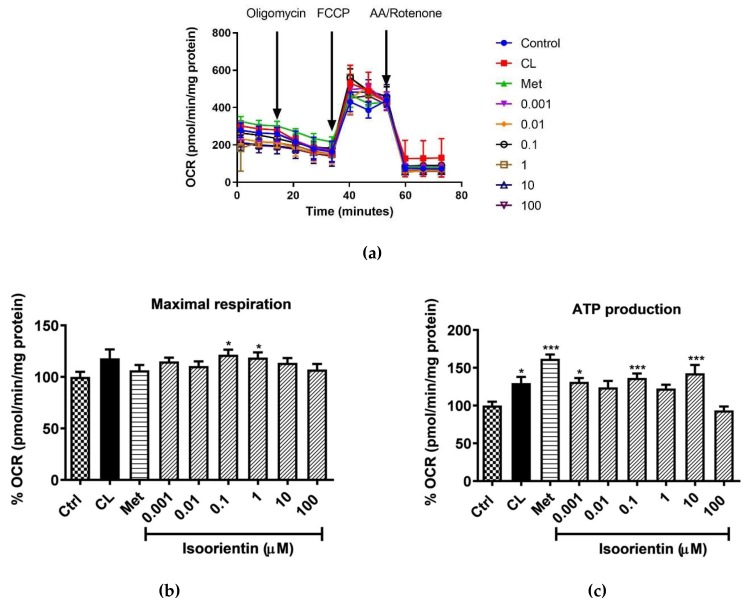
Isoorientin enhances mitochondrial respiration in fully differentiated 3T3-L1 adipocytes, (**a**) representative oxygen consumption rate (OCR) plot; (**b**) maximal respiration; and (**c**) ATP production. Matured 3T3-L1 adipocytes were treated with or without positive control CL-316,243 (CL 1), isoproterenol (Isopr ) used at 1 µM and various doses of isoorientin (0.001, 0.01, 0.1, 1, 10, and 100 µM) for 4 h; thereafter, mitochondrial respiration was measured using a Seahorse XF analyzer. Results are expressed as mean ± SEM of three independent experiments. * *p* < 0.05, *** *p* ˂ 0.001 versus normal control (Ctrl). The numbers 0.001, 0.01, 0, 1, 1, 10, and 100 represent the isoorientin concentration used in μM units.

The ability of isoorientin to enhance mitochondrial respiration in matured 3T3-L1 adipocytes was determined with the Seahorse analyzer, and the representative OCR plot is displayed in Figure 3a. The results showed that in terms of OCR, isoorientin increased the maximal respiration rate in all concentrations tested, with significance observed at doses of 0.1 and 1 µM (*p* < 0.5) (Figure 3b). After an oligomycin injection, positive controls CL and metformin, significantly enhanced ATP production by 32% and 62% (*p* < 0.5 and *p* < 0.001), respectively. All concentrations of isoorientin tested were able to increase ATP production, except for the highest dose (100 μM) tested (Figure 3c).

### 2.4. Isoorientin Increased AKT and AMPK Activation in Fully Differentiated 3T3-L1 Adipocytes

**Figure 4:**
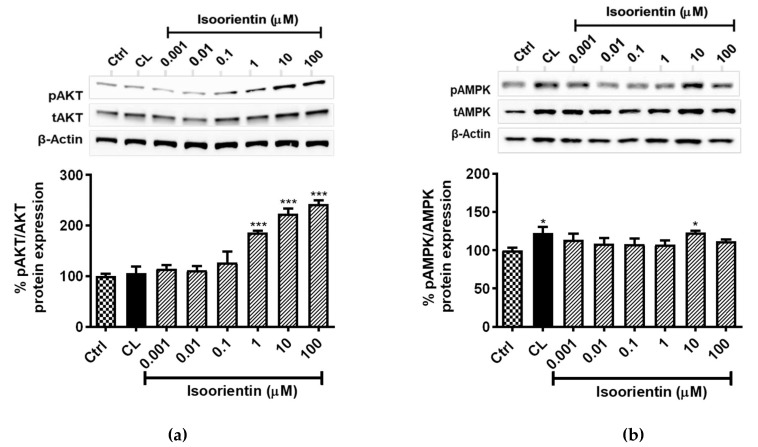
Isoorientin increased protein kinase B (pAKT), (**a**) 5′ AMP-activated protein kinase (pAMPK); and (**b**) protein expression in fully differentiated 3T3-L1 adipocytes. Matured 3T3-L1 adipocytes were treated with or without positive control CL-316,243 (CL), isoproterenol (Isopr) used at 1 µM and various doses of isoorientin (0.001, 0.01, 0.1, 1, 10, and 100 µM) for 4 hours; thereafter, proteins were extracted for Western blot analysis. Results are expressed as mean ± SEM of three independent experiments. * *p* < 0.05, *** *p* < 0.001 versus normal control (Ctrl).

It is increasingly acknowledged that AKT and AMPK pathways are well studied signaling mechanisms, associated with the regulation of lipid metabolism, which also impact mitochondrial function. The results showed that isoorientin doses ≥ 1 μM, strongly enhanced AKT phosphorylation (*p* < 0.001), by 86%, 123%, and 142%, respectively for 1, 10, and 100 µM, when compared to the untreated control (Figure 4a). In addition, isoorientin at a dose of 10 µM, significantly enhanced the phosphorylation of AMPK, at a level comparable to that of a positive CL control (22%, *p* < 0.05) (Figure 4b).

## 3. Materials and Methods 

### 3.1. Materials

3T3-L1 mouse embryonic fibroblasts (Cat# CL-173) were purchased from the American Type Culture Collection (Manassas, VA, USA). Dulbecco’s modified Eagle’s medium (DMEM), Dulbecco’s phosphate buffered saline (DPBS), penicillin/streptomycin and trypsin/versene were bought from Lonza Biowhitaker (Walkersville, MD, USA). Fetal bovine serum (FBS) were obtained from Gibco, Invitrogen (EU Approved, origin: South America). Isoorientin (≥ 98.0% purity), CL316243 (CL), dexamethasone (DEX), 3-isobutyl-1-methylxanthine (IBMX), insulin, oil red O (ORO), dimethyl sulfoxide (DMSO), Sodium biocarbonate (NaHCO_3_), as well as phenol red and glucose-free DMEM were procured from Sigma-Aldrich (St. Louis, MO, USA). The ViaLight^TM^ plus ATP kit was obtained from Lonza (Basel, Switzerland). Bradford and RC DC protein assay kits were bought from Bio-Rad Laboratories (Hercules, CA, USA). Protease and phosphatase inhibitor tablets were purchased from Roche (South San Francisco, CA, USA). Seahorse XF96 microplate plates, Seahorse XF Assay media, and Seahorse XF-Cell Mito Stress assay kits were purchased from Agilent (Santa Clare, CA, USA). Cell Signalling Technology (Beverly, MA, USA) supplied primary antibodies including AKT (cat# 9272S), p-AKT (Ser473) (cat# 9271S), AMPK (cat# 2532), p-AMPK (Thr172) (cat# 2535S). The housekeeping β-actin (cat# sc-47778), secondary antibodies goat anti-rabbit (cat# sc-2004), and IgG– horseradish peroxidase were purchased from Santa Cruz Biotechnology (Dallas, Texas, USA). All other reagents and chemical were from Sigma unless specified.

### 3.2. Methods

#### 3.2.1. Cell Culture and Differentiation of 3T3-L1 Adipocytes

Mouse 3T3-L1 pre-adipocytes were cultured in a growth medium (DMEM containing 10% FBS), in a humidified atmosphere of 37 °C and 5% CO_2_, as previously described [21]. Briefly, pre-adipocytes were seeded in a growth media at a density of 20,000 cells/mL, which allowed them to reach confluency within 3 days in 24-well plates for ORO staining, 96-well microplates for ATP and MTT assays, as well as 6-well plates for protein expression analysis. Briefly, the differentiation process was as follows: upon confluency (day 0), growth medium was substituted with adipocyte differentiation media (DMEM containing 10% FBS, 0.5 mM IBMX, 1 μM DEX, and l μg/mL insulin), for 2 days. On day 3, the medium was changed to insulin media or adipocyte maintenance media (DMEM containing 1 μg/mL insulin) for a further 2 days. From day 5, cells were maintained in the growth medium, until day 8. After day 8, cells were fully differentiated to mature adipocytes; thereafter, relevant assays were performed.

#### 3.2.2. Preparation of Compounds and Treatment

Isoorientin stock was prepared by dissolving 10 mg in 100% DMSO (1 mL) to prepare a concentrated solution of 22.30 mM, which was stored at −80 °C until further use. The initial stock was further diluted in media to yield working concentrations of 0.001, 0.01, 0.1, 1, 10, and 100 µM. All compounds, including isoorientin and two positive controls of pioglitazone and CL-316,243, were prepared by diluting the appropriate amounts of a stock solution in phenol red free DMEM (supplemented with 8 mM glucose, 3.7 g/L NaHCO_3_, and 0.1% (*w*/*v*) bovine serum albumin (BSA)), to yield the final working concentrations. To eliminate interference of DMSO in the bioactivity of compounds, the final DMSO in all experiments was <0.001%, as previously reported [22]. Briefly, the treatment process involved a nutrient deprivation step where differentiated adipocytes were cultured in phenol red free DMEM, without glucose and serum (supplemented with 3.7 g/L NaHCO_3_, 0.1% (w/v) BSA), for 30 min prior to the addition of the relevant treatment. Cells were subsequently treated with various concentrations of isoorientin (0.001, 0.01, 0.1, 1, 10, and 100 μM) in DMEM without phenol red (containing 8 mM glucose, 3.7 g/L NaHCO_3_, 0.1% (w/v) BSA), for 4 hours, in a humidified atmosphere of 37 °C and 5% CO_2_. Positive controls such as insulin (1 μM), metformin (1 μM), isoproterenol (10 μM), and CL-316,243 (10 μM), were also added for 4 hours [23]. Dose and time selection was based on previously published evidence from our lab, demonstrating that isoorientin is able to improve glucose uptake in C_2_C_12_ cells exposed to isoorientin for 3 h [24].

#### 3.2.3. ATP Content and Glucose Uptake Determination

After treatment for 4 hours, the ATP content, as a measurement of metabolic activity, was quantified using the ViaLight^™^ plus sample kit (Lonza, Basel, Switzerland), following the manufacturer’s protocol. Luminescence was measured using a BioTek^®^ FLx800 plate reader equipped with Gen 5^®^ software (BioTek Instruments Inc., Winooski, VT, USA). Glucose uptake was performed as previously described by [25] with slight modifications. Briefly, after 4 hours of treatment, adipocytes were incubated with DMEM without phenol red, containing 0.5 µCi/mL 2-deoxy-[^3^H]-d-glucose (Radiolabeled Chemicals, St Louis, MO, USA), for 15 min at 37 °C in 5% CO_2_. Subsequently, 2-deoxy-[^3^H]-d-glucose uptake was assessed by liquid scintillation, as described by [24]. Thereafter, 2-deoxy-[^3^H]-d-glucose uptake was assessed in the lysate by liquid scintillation (2220 CA, Packard Tri-Carbseries, PerkinElmer, Downers Crove, IL, USA). 

#### 3.2.4. Intracellular Lipid Content and Glycerol Determination

Intracellular lipid accumulation was determined using ORO, as described by [16]. After 4 hours of treatment, media was collected for glycerol, cells were stained with 0.7% (*v*/*v*) ORO, and lipid droplets were visualized using a Nikon inverted fluorescent microscope (Nikon eclipse Ti; Nikon, Japan), equipped with a camera (Nikon DXM1200) and NIS-Element imaging software. Stained droplets were dissolved with isopropanol and quantified by spectrophotometric analysis at 570 nm using a BioTek^®^ ELx800 plate reader equipped with Gen 5^®^ software for data acquisition. The lipid content was normalized to cell density (crystal violet staining measured at 570 nm). Moreover, the amount of free glycerol in the collected cell culture medium was ascertained using the fluorescent Glycerol Release Assay kit from Bio vision Inc. (Milpitas, CA, USA), according to the manufacturer’s protocol. Free glycerol content was normalized to cell density (crystal violet measured at 570 nm).

#### 3.2.5. Assessing Mitochondrial Bioenergetics

Mitochondrial bioenergetics were assessed by determining OCR, using a Mito Stress assay kit and XF96 Extracellular Flux analyzer (Agilent, Santa Clara, CA, USA), as described previously [26], with some modifications. Briefly, 3T3-L1 adipocytes were seeded in 96-well XF microplates at 10,000 cells per well, which reached confluence after 24 hours. Subsequently, cells were differentiated and treated as previously described in the cell differentiation and treatment section; however, the Seahorse assay was performed on day 5 of differentiation. Briefly, after treatment, cells were washed twice with 180 μL of pre-warmed XF assay medium (XF base medium containing 8 mM glucose, 2 mM glutamine, and 1 mM sodium pyruvate at pH 7.4) and incubated with the same volume of XF assay medium at 37 °C in a non-CO_2_ incubator for 1 h, to equilibrate temperature and pH prior to OCR measurement. ATP production and maximal respiration were determined by treating the cells with 1 μM oligomycin and 0.75 μM carbonyl cyanide-4-trifluoromethoxy-phenylhydrazone (FCCP). After the assay, the Bradford protein assay was used to determine protein concentration, and then OCR (pmol/min) was normalized relative to the protein content. OCR was reported as absolute rates, i.e., pmol/min/mg.

#### 3.2.6. Western Blot Analysis

Western blot analysis was performed as described by [25] with some modifications. In brief, the primary antibodies AMPK (1:1000), p-AMPK (Thr172) (1:800), AKT (1:1000), p-AKT (Ser473) (1:1000), and the reference protein β-actin (1:1000) were used and incubated at 4 °C overnight. The following day, the respective HRP-conjugated secondary antibodies goat anti-mouse or goat anti-rabbit (1:4000), were applied for 90 min at room temperature. Protein bands were visualized using the ChemiDoc^™^ MP system (BioRad, Cressier, Switzerland), and Image Lab software version 5.2.1 was used for analysis.

#### 3.2.7. Statistical Analysis

All results are expressed as the means ± SEM of three independent biological experiments. The significance of difference was determined using one-way analysis of variance (ANOVA), followed by Tukey’s post hoc test using GraphPad Prism version 6.07 (GraphPad Software Inc., San Diego, CA, USA). Results were considered significant at *p* < 0.05.

## 4. Discussion

Murine 3T3-L1 adipocytes remain a well-established and suitable model for investigating adipocytic lipid metabolism and obesity in vitro. This is evidenced by an increasing number of studies that utilize this model to screen for anti-obesity properties of plant extracts and their natural products [14,27]. During the differentiation of 3T3-L1 pre-adipocytes, the changes in cell size and number are known to be regulated by adipogenic transcription factors, including CCAAT/enhancer binding protein (C/EBPs) and PPARγ [28]. These transcription factors are known to promote adipogenesis in various cell models of obesity, as reviewed elsewhere [29]. Furthermore, AKT and AMPK signaling pathways are also known to play a crucial role in the regulation of adipogenesis. In fact, the activation of the AKT pathway promotes adipogenesis by upregulating PPARγ and C/EBPα, hence increasing lipid accumulation in 3T3-L1 adipocytes [30]. Activation of AKT can also promote insulin-dependent glucose uptake by mainly promoting translocation of glucose transporters, as observed in skeletal muscles exposed to palmitate [31]. On the other hand, activation of the AMPK pathway, a key sensor and regulator of cellular energy, inhibits adipogenesis, in part by blocking the expression of PPARγ in experimental models of obesity [32,33]. AMPK is also implicated in insulin-independent glucose uptake, and this process is revised in detail by Hayley et al. [34], which looks at various experimental models of insulin resistance. Importantly, as an integral process in controlling the process of adipogenesis, these pathways are known to greatly impact mitochondrial function in conditions of obesity. Consistently, activation of AMPK has been associated with increased β-oxidation, including the process of mitochondrial energetics [26]. Therefore, the treatments that reduce lipid accumulation, while enhancing energy metabolism via the aforementioned mechanisms, may provide an effective preventive or therapeutic approach for obesity.

The current study reports on essential evidence on the impact of isoorientin on lipid accumulation in an in vitro model of obesity. Such evidence includes, the observed concentration-dependent beneficial effects of isoorientin on blocking lipid storage, enhancing glycerol release, as well as improving metabolic activity and mitochondrial function [35]. Although the observed beneficial effects of isoorientin on these cultured 3T3-L1 adipocytes were concentration-independent, it was clear that doses between 1 and 10 μM presented with enhanced effects compared to the untreated control, and their activity was comparable to tested positive controls of CL, isoproterenol and metformin. Interestingly, the beneficial effects reported with the conducted bioassays of lipid accumulation and mitochondrial respiration showed consistent results in activating some of the crucial pathways involved in the regulation of these processes, especially isoorientin doses ≥ 1 μM. For instance, higher doses of isoorientin significantly enhanced the expression of AKT and AMPK, which could have inhibited lipogenesis by increasing energy expenditure. These results also link well with the implication of AMPK in the regulation of mitochondrial energetics and oxygen consumption in 3T3-L1 adipocytes, as previously reported [35]. Although other studies, or rather findings using plants containing this flavone, have been investigated for their anti-obesity properties [6,36], there is generally limited knowledge on the impact of isoorientin on lipid accumulation in matured adipocytes, especially its role in mitochondrial function and regulatory mechanisms.

Furthermore, results from the current study also point to the importance of dose-dependent considerations when investigating the bioactivity of diverse phytochemical compounds, such as isoorientin. Certainly, other researchers [37] have demonstrated that, depending on time, some polyphenols are more stable in human plasma than in cell culture conditions, which may be correlated to improved polyphenol–protein interactions in vivo. Thus, more research targeting the impact of dose and time response of isoorientin in vivo is necessary to better understand its pharmacokinetic profile. This could also highlight the limited investigations that have assessed the bioactivity properties of isoorientin against metabolic complications using in vivo models. Nevertheless, plant extracts containing this flavone have been shown to have an enhanced effect in lowering blood glucose levels, in protecting against β-cell damage, and improving the antioxidant status of diabetic rats [38,39]. Consistently, others show that plants, such as buckwheat containing this flavone, can ameliorate the insulin resistance and improve energy and glucose metabolism in fructose-diet exposed or estrogen-deficient rats [40,41]. Thus, these findings further highlight the potential and important role that isoorientin can play in energy regulation in conditions of enhanced lipid accumulation. However, additional studies are required to assess the impact of this dietary flavone on key adipocyte browning specific markers, such as peroxisome proliferator activated receptor-alpha (PPARα), peroxisome proliferator-activated receptor gamma coactivator 1 α (PGC1α), and sirtuin (SIRT1), using established in vivo models.

## 5. Conclusions

This study advocates the anti-obesity effect of isoorientin and describes some of the underlying mechanisms involved in its observed bioactivity in fully differentiated 3T3-L1 adipocytes. It also provides the first evidence that isoorientin could enhance mitochondrial respiration by stimulating ATP production and the oxygen consumption rate, an important process linked with efficient mitochondrial function. However, further investigations on the expression of key adipocyte browning specific markers, are still required to provide a better understanding of the underlying molecular mechanisms. Consistently, in vivo studies exploring dose and time response to isoorientin treatment, are needed to improve our understanding of its pharmacokinetic profile.
